# Targeting osteosarcoma with canine B7-H3 CAR T cells and impact of CXCR2 Co-expression on functional activity

**DOI:** 10.1007/s00262-024-03642-4

**Published:** 2024-03-30

**Authors:** Jennifer W. Cao, Jessica Lake, Renata Impastato, Lyndah Chow, Luisanny Perez, Laura Chubb, Jade Kurihara, Michael R. Verneris, Steven Dow

**Affiliations:** 1 Department of Microbiology, Immunology, and Pathology, Flint Animal Cancer Center, College of Veterinary Medicine and Biomedical Sciences, Colorado State University, Campus Delivery 1678, Fort Collins, CO USA; 2 Department of Clinical Sciences, Flint Animal Cancer Center, College of Veterinary Medicine and Biomedical Sciences, Colorado State University, Fort Collins, CO USA; 3 Department of Pediatrics, Center for Cancer and Blood Disorders, University of Colorado and Children’s Hospital of Colorado, Research Complex 1, North Tower 12800 E. 19th Ave. Mail Stop 8302, Room P18-4108, Aurora, CO 80045 USA

**Keywords:** Dog, Immune, Cytokine, Cancer, Cytotoxicity, Mouse

## Abstract

The use of large animal spontaneous models of solid cancers, such as dogs with osteosarcoma (OS), can help develop new cancer immunotherapy approaches, including chimeric antigen receptor (CAR) T cells. The goal of the present study was to generate canine CAR T cells targeting the B7-H3 (CD276) co-stimulatory molecule overexpressed by several solid cancers, including OS in both humans and dogs, and to assess their ability to recognize B7-H3 expressed by canine OS cell lines or by canine tumors in xenograft models. A second objective was to determine whether a novel dual CAR that expressed a chemokine receptor together with the B7-H3 CAR improved the activity of the canine CAR T cells. Therefore, in the studies reported here we examined B7-H3 expression by canine OS tumors, evaluated target engagement by canine B7-H3 CAR T cells in vitro, and compared the relative effectiveness of B7-H3 CAR T cells versus B7-H3-CXCR2 dual CAR T cells in canine xenograft models. We found that most canine OS tumors expressed B7-H3; whereas, levels were undetectable on normal dog tissues. Both B7-H3 CAR T cells demonstrated activation and OS-specific target killing in vitro, but there was significantly greater cytokine production by B7-H3-CXCR2 CAR T cells. In canine OS xenograft models, little anti-tumor activity was generated by B7-H3 CAR T cells; whereas, B7-H3-CXCR2 CAR T cells significantly inhibited tumor growth, inducing complete tumor elimination in most treated mice. These findings indicated therefore that addition of a chemokine receptor could significantly improve the anti-tumor activity of canine B7-H3 CAR T cells, and that evaluation of this new dual CAR construct in dogs with primary or metastatic OS is warranted since such studies could provide a critical and realistic validation of the chemokine receptor concept.

**Supplementary Information:**

The online version contains supplementary material available at 10.1007/s00262-024-03642-4.

## Introduction

Chimeric antigen receptor (CAR) T cells are engineered to target specific tumor antigens to eliminate tumor cells following adoptive cell therapy [1]. Most notably, CD19 targeted CAR T cell therapy has revolutionized the treatment of B cell malignancies (leukemias and lymphomas) achieving complete remissions in up to 80% of patients with previously treated, advanced relapsed or refractory B cell acute lymphoblastic leukemia [2–4]. However, CAR T cell therapy is notably less effective for treatment of solid tumors, for reasons that are still being elucidated [5]. The proposed major barriers to effective CAR T cell therapy for solid tumors include the immune suppressive effects of the tumor microenvironment (TME), the failure of adequate CAR T trafficking, tumor heterogeneity and target antigen downmodulation [5, 6]. To date, multiple approaches have been assessed to overcome these barriers, but successes realized in murine tumor models have largely not been translated to date to human clinical trials [7, 8].

Osteosarcoma (OS) is an aggressive tumor primarily affecting children and adolescents, with 15-20% of patients having metastatic disease at the time of diagnosis [9, 10]. Patients with metastatic disease have a 5-year survival rate of less than 20% and clinical outcomes for metastatic patients have not improved in over 4 decades [9, 11, 12]. Therefore, patients with OS represent an attractive population for evaluating new strategies in CAR T development. The checkpoint molecule B7 homolog 3 (B7-H3; CD276) is expressed normally at low levels on human and mouse myeloid cells (dendritic cells, monocytes, and macrophages) and most normal tissues [13, 14]. However, B7-H3 is overexpressed by many different solid tumors, including OS [15–17]. Other studies have shown that B7-H3 drives differentiation of osteoblasts during embryogenesis and plays a role in bone mineralization [18, 19]. High levels of B7-H3 expression in patients with OS has also been correlated with poor outcomes, more rapid metastatic progression, and enhanced immune evasion, providing a strong rationale for targeting B7-H3 is a promising target [20, 21].

Two therapeutic monoclonal antibodies (8H9 and MGA271) targeting human B7-H3 have been evaluated clinically for treatment in human solid tumors including non-small cell lung cancer, triple negative breast cancer, brain and CNS tumors and sarcomas, but these trials revealed only marginal efficacy. Although clinical results with the 8H9 mAb (^131^I--omburtamab) showed increased survival time in neuroblastoma patients [22], this drug was ultimately not approved by the FDA; whereas, the MGA271 trial prematurely closed due to multiple fatal events [23–25]. These early studies paved the way for the evaluation of B7-H3 as a target for CAR T cells. Studies of B7-H3 CAR T cells in NOD*-scid* IL2Rgamma^null^ (NSG) mice demonstrated anti-tumor activity against xenograft models of high B7-H3 expressing prostate cancer, pulmonary giant cell carcinoma, subcutaneous ovarian carcinoma and osteosarcoma [17, 26–29]. Phase I clinical trials of B7-H3 CAR T cells have been completed in adult patients with advanced solid tumors, including OS, neuroblastoma, gastric cancer, and lung cancer (NCT04864821) and in pediatric patients with recurrent or refractory tumors including OS (NCT04483778), with the best response in the latter trial reported as stable disease in 3 out of 9 pediatric patients [30]

To help advance the evaluation of B7-H3 CAR T cells in the treatment of pediatric OS, the canine spontaneous tumor model offers advantages of an intact immune system and an appropriate body size for realistic CAR T cell dosing. Unlike the case with mouse models for OS; dogs are exposed to the same environmental factors as humans, the tumor develops in similar locations [31, 32], and the tumor in dogs harbors mutations that closely resemble those present in human OS [33, 34]. Lastly, the TME of canine OS shows many immune cell signatures present in the human OS TME [35], suggesting that responses to new immunotherapeutics in dogs may more accurately predict human responses to similar immunotherapeutics [36]. In a previous study, canine B7-H3 CAR T cells demonstrated *in vitro* activity against canine OS cell lines and high levels of B7-H3 expression by the cell lines and tumor tissues was reported [37–39]. Importantly, the canine B7-H3 CAR T cells did not trigger adverse events when administered to healthy beagle dogs [39].

Failure of CAR T cell trafficking is a major proposed mechanism for ineffective CAR T cell treatment in solid tumors [5, 7]. Genetic modification and *ex vivo* expansion of CAR T cells have been shown to downmodulate chemokine receptors, thereby impairing T cell homing mechanisms [40, 41]. Therefore, the overexpression of chemokine receptors such as CXCR2 has been used to improve CAR T cell migration to tumor tissues [42–44]. CXCR2 is of particular interest because its primary ligand CXCL8 exerts strong pro-tumor effects through recruitment of myeloid derived suppressor cells (MDSCs) [45], enhancement of angiogenesis [46] and increased tumor metastases [44, 47]. Addition of CXCR2 expression by GPC3-targeted CAR T cells has previously been shown to increase homing and produce greater *in vivo* anti-tumoral effects in a mouse xenograft model of hepatocellular carcinoma (HCC) [42]. In addition, augmentation of CXCR2 expression to CD70 CAR T cells have also increased CAR T cell migration and persistence within tumors in mouse models of glioblastoma, ovarian and pancreatic cancer [43].

In the current study we expanded the scope of the original canine B7-H3 CAR T cell studies [39] to evaluate expression of B7-H3 using a larger panel of canine OS tumor cell lines and biopsy tissues. In addition, the current study included *in vitro* and *in vivo* characterization of a dual B7-H3 CAR construct co-expressing CXCR2. We report here that B7-H3 was overexpressed in the majority of canine OS tumors evaluated and by all the canine OS cell lines tested. Importantly, a canine OS xenograft model was used successfully to demonstrate the *in vivo* superiority of a dual B7-H3-CXCR2 CAR construct for tumor control. These findings thus provide strong rationale for evaluating canine B7-H3-CXCR2 CAR T cells in preclinical studies in dogs with primary or metastatic OS to help validate the approach for treatment of pediatric OS.

## Materials and methods

### Animals

NOD-SCID-gamma (NSG) mice were purchased from Jackson Laboratories (Bar Harbor, ME). CAR T cells were generated from blood collected by jugular venipuncture from dogs with osteosarcoma, following owner permission. All animal studies were approved by the Colorado State University Animal Care and Use Committee.

### Cell lines

The human erythroleukemia cell line K562 was stably transduced to overexpress both FcyRII (CD32) and canine CD86 as a means of generating artificial antigen presenting cells (aAPCs), as previously described, and the cells were kindly provided by Dr. Nicola Mason (University of Pennsylvania, School of Veterinary Medicine) [48, 49]. These aAPCs were conjugated with anti-canine CD3 (clone CA17.2A12, Bio-Rad) and anti-canine CD28 (clone 5B8, Invitrogen) at a 1:1 ratio as previously described [48]. Canine osteosarcoma cell lines were provided by the FACC cell line repository and were previously validated [50]. All cell lines were cultured in cell culture medium containing DMEM with 10% fetal bovine serum (Corning), 2 mM L-glutamine (Gibco), 100 U/ml penicillin and 100 ug/ml streptomycin (Gibco). All cell cultures were maintained at 37C and 10% CO_2_.

### CAR constructs

The B7-H3 CAR construct was shared with the University of Colorado Verneris lab by Michael Jensen, MD (Fred Hutchinson Cancer Center, Seattle, WA). This lentiviral construct contained a B7-H3 single-chain variable fragment (scFv), an IgG4 and CD28 transmembrane domain, a 4-1BB co-stimulatory molecule and the CD3ζ domain. The B7-H3 CAR construct used was cloned into a retroviral backbone containing a 5' LTR promoter using standard PCR-based cloning methods to create the B7-H3 CAR construct. The retroviral backbone we used was a gift from Dr. Anandani Nellan (National Cancer Institute, Bethesda, MD). The B7-H3-CXCR2 CAR construct was created first by extracting native CXCR2 RNA from human neutrophils, conversion to cDNA by reverse transcriptase (Bio-Rad iScript cDNA Synthesis kit), and amplification of the CXCR2 sequence. The CXCR2 DNA fragment was inserted into the B7-H3 CAR construct using PCR cloning methods by enzymatic cleavage and ligation.

### Retroviral vector production

To produce the retroviral vectors used in these studies, 293GP cells were transduced with plasmids for B7-H3 and B7-H3-CXCR2, using lipofectamine (InVitroGen) following manufacture recommended protocol and then viral supernatant was collected at 24 and 48 hours after transduction. The retroviral stocks were flash frozen in dry ice and placed in -80 ˚C until use.

### Generation of anti-canine CD3/CD28 magnetic beads

Mouse anti-canine CD3 (clone CA17.2A12, Bio-Rad) and mouse anti-canine CD28 (clone 5B8, Invitrogen) antibodies were conjugated to magnetic tosyl-activated Dynabeads (Life Technologies) as previously established at a 1:1 antibody ratio [48, 51]

### Canine PBMC isolation and T cell activation

Blood from dogs with recently diagnosed sarcoma (prior to any treatment) was collected by jugular venipuncture by Flint Animal Cancer Center oncology clinical service staff. Peripheral blood mononuclear cells (PBMCs) were isolated from EDTA-anticoagulated blood using Ficoll-plaque (VWR) density gradient centrifugation. The PBMCs were plated at a density of 2.0 x 10^6^/mL in 6-well tissue culture plates to allow adherence of monocytes for 90 minutes at 37 ˚ C and 10% CO_2_. The non-adherent, monocyte depleted lymphocytes (PBL) were gently removed using complete T cell media (cTCM), which consisted of DMEM with 10% FBS and 100 U/ml penicillin and 100 ug/ml streptomycin (Gibco), 2 mM L glut, 1x essential amino acids (Gibco), 1x non-essential amino acid (Gibco), 0.075% bicarbonate solution, 10 mM HEPES, and 55 uM 2-mercaptoethanol (Gibco). The PBL were plated at a concentration of 1.0 x 10^6^/mL in cTCM supplemented with 30 ng/mL rhIL-21(PeproTech) and 100 IU rhIL-2 (PeproTech). In some studies, additional cytokines including 5 ng/mL rhIL-7 (PeproTech) and rhIL-15 (PeproTech) were also added. The T cells were activated using either tosyl-activated Dynabeads (Life Technologies) conjugated with canine anti-CD3/CD28 antibodies at a ratio of 3:1 beads to PBLs, or with 5 ug/ml PHA, or with 1:1 ratio of anti-CD3/CD28 conjugated aAPCs. The activated T cells were incubated at 37˚ C for 3 days before transduction with CAR containing retroviral vectors.

### CAR T cell Transduction

Canine CAR T cells were generated according to previously published protocol with a few modifications [51]. Non-tissue culture treated plates were coated with 24 µg/mL RetroNectin (Takara Bio) according to the manufacture’s protocol. Frozen B7-H3 retroviral supernatant was thawed in a 37 °C water bath and aliquoted into RetroNectin coated 6-well plates at 2 ml/well and centrifuged at 32 °C for 2.5 hours at 2,000 xg. Viral supernatant was discarded after centrifugation and activated PBLs were plated onto retroviral bound plates at a concentration of 5.0 x10^5^ cells/mL and 2 mL per well was added and incubated overnight. The CAR T cells were transduced twice before being rescued from virus and put into fresh cTCM with fresh cytokines. Half of the medium was changed, and fresh cytokines are added every 2-3 days. The CAR T cells were used for analysis on day 10–14 of culture.

### Flow cytometry

Expression of B7-H3 on PBMCs and OS cell lines was analyzed using anti-B7-H3-APC antibody (clone 7-517, eBioscience), along with an isotype control mouse IgG2a antibody (ThermoFisher, cat# 02-6200). The CAR T cells were further characterized using anti-canine CD5-FITC (cloneYKIX322.3, Bio-Rad), anti-canine CD8-alexa Fluor 647 (clone YCATE55.9, Bio-Rad), anti-canine CD4-Pacific Blue (clone YKIX302.9, Bio-Rad), anti-CD62L (clone FMC46, Bio-Rad), anti-CD45RA (clone CA21,4B3, Leukocyte Antigen Biology Laboratory). CAR expression was also assessed using biotinylated recombinant Protein L (ThermoFisher, cat# 29997) with secondary streptavidin-PE (eBioscience). Expression of human CXCR2 was assessed using anti-CXCR2-PEcy7 (clone 5E8, Biolegend). Samples were processed on a Beckman Coulter Gallios flow cytometer and data were analyzed using Flowjo v10.

### CAR T cell activation and cytokine release

Tumor target engagement by B7-H3 CAR T cells was assessed using canine OS cell lines. Non-transduced T cells from the same animals were used as controls for non-specific tumor cell killing. As one measure of B7-H3 CAR T cell activation, cytokine release was assessed after 24 hours of co-culture with target cells, using specific ELISA assays. For measuring cytokines, canine OS cell lines were plated at 5 x 10^3^ cells/ well in cTCM in a 96 well flat bottom plates (ThermoFisher) and incubated for 24 hours at 37 °C. T cells were washed 3x with 10 mL cTCM media to ensure no recombinant IL-2 was carried over into the tumor co-culture assays. The CAR T cells were then plated 1:1 effector to target (E:T) ratios (using total T cell numbers for calculation) with canine OS cell lines to a final volume of 250 µl/ well. Co-cultures were incubated 24 hours, 100 µL/well of supernatant was then collected for cytokine assay. Cytokines were assayed using canine specific ELISA kits, including IFNγ ELISA (R&D Systems) and canine IL-2 (R&D Systems), according to manufacturer’s directions. Chemokine secretion was also measured in co-culture assays. Cell lines were plated at a density of 5 x 10^4^ cells per well in a 24 well plates in 2 mL medium /well. Medium was changed 24 hours after the tumor cells had fully adhered to the plastic. Supernatants were collected 4 days later, supernatant was used in technical triplicates in a canine CXCL8 ELISA (R&D Systems), according to manufacturer’s protocol.

### CAR T cell freezing

Cells were washed and pelleted before freezing. Cells were resuspended in freezing medium, which consisted of DMEM with 10% FBS, and 10% Dimethyl Sulfoxide (DMSO). After suspension in freezing medium, the cells were aliquoted into 2 mL cryovials at maximum concentration 5.0 x 10^6^/vial and frozen at -80 °C in cell freezing containers (ThermoFisher). To recover frozen cells, B7-H3 CAR vials were thawed in a 37 ˚C water bath, washed with complete medium and incubated at 37 ˚C for 48 hours in cTCM medium with cytokines matching their initial expansion culture before use in any *in vitro* assays.

### CAR T cell migration assay

Migration assays were used to assess the recognition of canine CXCL8 by the huCXCR2 expressed on the dual CAR T cells. Migration was assessed using 8 µm transwell plates (Corning), with the lower chamber containing 25 ng/ml recombinant canine CXCL8 (R&D Systems) in serum free DMEM. Migration controls included negative controls (medium only) and positive controls (DMEM with 10% FBS). Prior to addition to the top chamber the CAR T cells were washed twice. Plates were incubated at 37˚ C for 4 hours and then the cells in the bottom well were collected and enumerated by hemocytometer.

### Tumor cell cytotoxicity assay

The ability of CAR T cells to kill target cells was assessed using an Incucyte system (Sartorius). Target cells (Abrams OS cells transduced to express red fluorescent protein) were seeded in a 96-well flat bottom plates (Corning) at 5.0x10^3^ cells/well and incubated for 24 hours to allow for adherence. In other studies, with untagged canine OS cell lines, the cells were first labeled with apoptotic marker caspase3/7-GFP (Sartorius) according to manufactures instructions to detect induction of apoptosis. Effector CAR T cells were added at several effector to target (E:T) ratios (1:1, 1:5 or 1:20) using total T cell count. Target cell lysis or apoptosis was assessed using a live cell imaging system (Incucyte). Cell images were captured every 2 hours for 48 hours, and data analyzed using Incucyte software.

### B7-H3 immunohistochemistry

Expression of B7-H3 by osteosarcoma tumors and normal dog lymph node was assessed using anti-B7 H3 antibody (clone RBT-B7H3, BioSB) by immunohistochemistry using 4 um sections from formalin fixed tumor sections (FFPE). Staining for normal dog tissues spleen and liver were performed with B7-H3 antibody (clone 7-517) on tissues fixed in zinc oxide (BD Biosciences). Immunohistochemistry on FFPE sections was performed as previously described by Zhang et al [39]. Images were analyzed using ImageJ software to quantitate the density of B7-H3 antigen expression [52]. An expression score was assigned a value of 0-300 calculated by determining the percentage of positive cells X the intensity of staining. n=5 of 20x magnification random area images were used for expression scoring. Sections were assigned a score of high>200, intermediate 200-100 or low <100 B7-H3 expression [53].

### RNA sequencing

Transcriptomic analysis of canine CAR T cells was done as described previously [54]. Cells were isolated after 10 days of expansion following transduction, and RNA was extracted with an RNeasy mini kit (Qiagen, Hilden Germany). The mRNA was sequenced at Novogene (San Diego) using an Illumina platform. Samples were tested for quality control by Agilent 2100 Bioanalyzer system and by agarose gel electrophoresis. Libraries were generated using SMART-Seq v4 3’ DE Kit (Takara Bio, Japan), then sequenced on an Illumina HiSeq platform for 150 base pair paired end reads at 4.0e7 raw reads per sample. Raw reads were filtered by removing reads containing adapter and reads containing *N*>10% and for Phred scores >30. The filtered reads were analyzed using Partek Flow software version 7.0. Filtered reads were aligned with STAR 2.73a CanFam3.1 genome assembly. Aligned reads were annotated and counted using HTseq [55]. Downstream analysis of raw count data was done in R (v3.6.1) and normalized using RUVseq package RUVr function [56, 57]. Differentially expressed genes were identified using Deseq2 R package [58] requiring a *P* value cutoff of 0.05 and a minimum log_2_ fold change of 1.5. Heatmaps were generated in R using “ComplexHeatmap” package [57]. Volcano plots were generated in R using “EnhancedVolcano” package [59]. Raw data including fasq files have been deposited in GEO (Gene Expression Omnibus) under accession number GSE247355.

Gene Set Enrichment Analysis (GSEA) was performed using normalized gene count to determine biological pathways differentially enriched between B7-H3-CXCR2 CAR T cells and B7-H3 CAR T cells [60, 61]. GSEA analysis was performed using publicly available gene sets housed in the Molecular Signatures Database (MsigDB) [62] using “h.all.v2023.1.Hs.symbosl.gmt”, “c5.go.bp.v2023.1.Hs.symbols.gmt, “c5.go.mf.v2023.1.Hs.symbols.gmt” and “c7.all.v2023.1.Hs.symbols.gmt” as reference gene sets. Statistically significant pathways were set at P value <0.05 and false discovery rates q <0.05.

### Canine OS xenograft model for assessment of CAR T cell activity.

NOD-SCID-gamma (NSG) mice (Jackson Laboratories) (n = 5-7 per group) were injected with 1.5 x10^5^ canine OS cells (Abrams-luciferase transfected) in 30% Matrigel (Corning) subcutaneously in the right flank. Tumor implantation was confirmed through IVIS imaging before treatment. Canine CAR T cells, either B7-H3 CAR or B7-H3-CXCR2 CAR, were administered to the mice by intravenous tail vein injection on day 3 after tumor implantation at 5.0 x10^6^ CAR T cells/mouse. CAR T transduction percentage was verified by flow cytometry the day of injection. Engrafted tumors were monitored with serial IVIS imaging and measurement with calipers when tumors had grown large enough to palpate. Control groups were given no treatment. To assess CAR T cell survival in vivo, in a subset of mice (*n* = 3 per group) blood was collected by tail vein on day 14 after CAR T cell injection to quantify circulating cells. Tumor diameter (calipers) and tumor luciferase expression (IVIS imaging) was measured 2-3 times per week, and mice were euthanized after meeting pre-determined endpoints. Personnel monitoring tumor growth were unaware of group assignments.

### Western blot for B7H3 cross reactivity

A standard Western blotting protocol from Bio-Rad was followed. Briefly, canine OS cell line Abrams and human rhabdomyosarcoma cell line RH30 were collected and washed with 1x PBS. Samples were prepared under non-reducing, denaturing conditions and 20 ug total protein was loaded into a gel (Bio-Rad, 4561084). B7-H3 monoclonal antibody (Invitrogen, MA5-15693) was used to probe for B7-H3 protein, followed by peroxidase-conjugated donkey anti-mouse secondary antibody (Jackson, 715-035-150).

### Statistical analysis

For the comparison of mean values between three or more groups normality was tested with a Shapiro–Wilk test, and normally distributed data were compared using ordinary one-way ANOVA. Data that was not normally distributed was analyzed using Kruskal–Wallis test followed by multiple comparison Dunn’s test. For comparisons between two groups, an unpaired two-tailed T test was performed. All statistical tests were performed in Graph Pad Prism 8 software (Boston, MA, USA).

## Results.

### Optimization of CAR T cell culture and expansion conditions

**Fig. 1:**
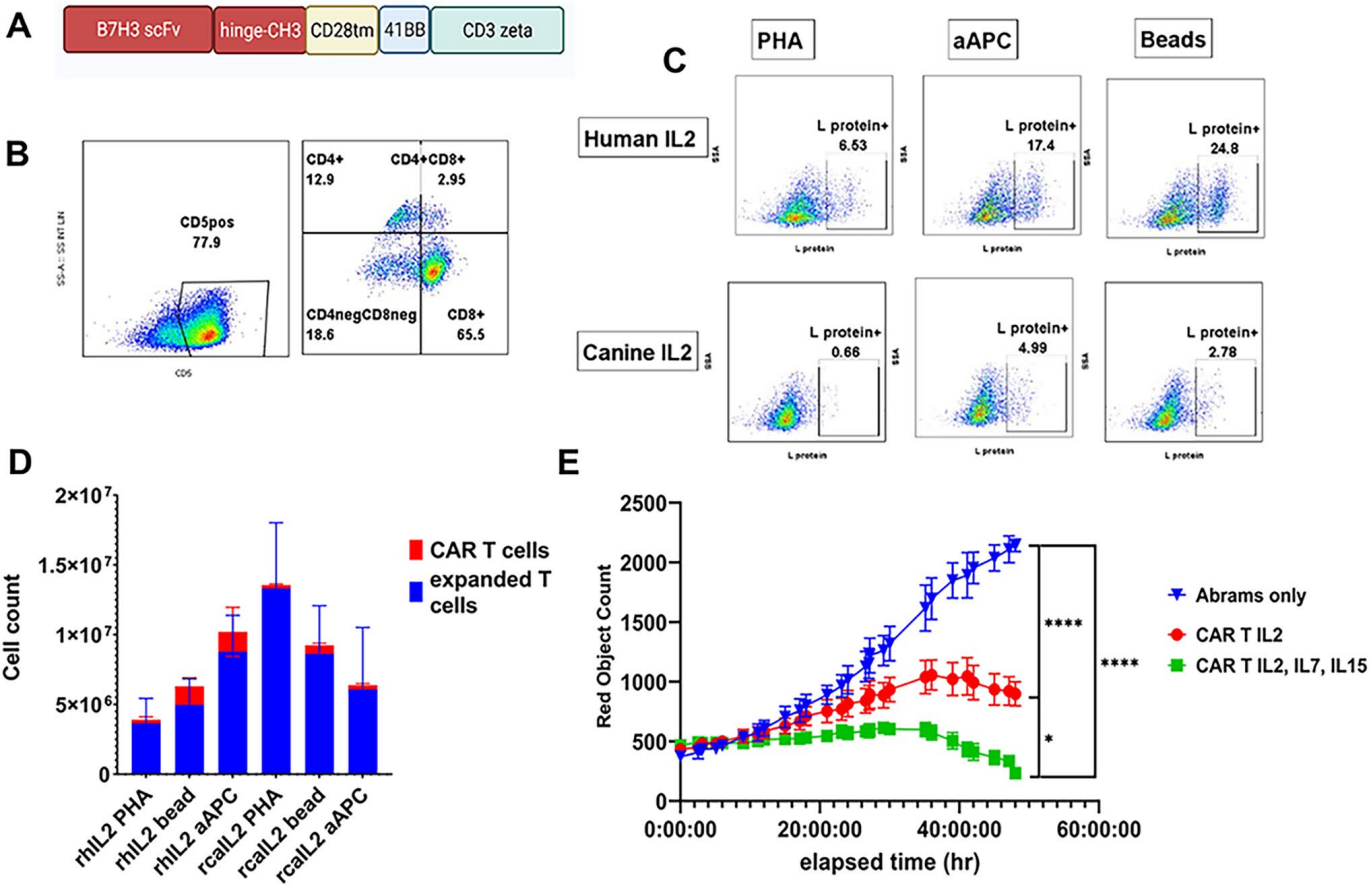
*Optimization of cell culture conditions for generating canine CAR T cells*. The impact of cell culture conditions, including T cell activation and T cell expansion, were evaluated to identify the optimal conditions. **A** Diagram of human B7-H3 CAR construct used to generate canine B7-H3 CAR T cells. In **B**, flow cytometry was used to determine expression of canine CD5 + T cell CD4 and CD8 subsets by day 10 CAR T cell cultures. In **C** the relative CAR T cell transduction efficiency was determined by immunostaining with recombinant L-protein. Canine T cells were activated with PHA, CD3/CD28 beads, or aAPCs, and expanded in rhIL-2 (top row) or rcIL-2 (bottom row), and the percentage of L-protein positive cells determined by flow cytometry. In **D**, CAR T cells were generated from *n* = 6 dogs (3 healthy, 3 osteosarcoma), and the mean ± SEM cell numbers and percentages of CAR T cells (red) and non-transduced T cells (blue) determined for each of the culture activation and expansion conditions (x-axis) determined, illustrating greatest CAR T generation when rhIL-2 was used for expansion. **E** Representative CAR T cell cytotoxicity study, using the canine OS cell line Abrams as the target, as described in Methods. Target killing over time determined for B7-H3 CAR T cells cultured in IL-2 + IL-21 (red) or IL-2 + IL-21 + IL-7 and IL15 (green), demonstrating superior killing activity of CAR T cells expanded in the 4-cytokine cocktail. Assays were run in triplicate, and statistical significance was determined using Dunn’s multiple means comparison test; *P < 0.05, *****, *P* < 0.0001, *ns* not significant

Initial studies were performed to optimize T cell expansion and viral transduction for optimal generation of CAR T cells. A second-generation B7-H3 CAR construct and a retroviral gene delivery system was used to generate canine B7-H3 CAR T cells (Fig. 1A). The first studies involved evaluation of best activation methods to expand T cells and were done using PBMC obtained from three healthy dogs and three dogs with OS. The activation conditions compared activation with anti-canine CD3/CD28 conjugated beads, PHA[63]or anti-canine CD3/CD28 conjugated aAPCs [48].

Next, we evaluated the impact of using human or canine recombinant IL-2 on canine CAR T cell expansion and function. The transduced cells were initially all cultured with recombinant human IL-21 on the first day [48]. Cytokines tested for CAR T cell expansion efficiency included recombinant human IL-2 (rhIL-2) (PeproTech) and recombinant canine IL-2 (rcaIL-2) (R&D Systems). Readouts included percentage of CD5+ canine T cells expanded from PBMCs, ratio of CD4+ and CD8+ T cells and percentage of CAR T cells as assessed by flow cytometry (Fig. 1B, C). These studies revealed that CAR T cells consisted of both CD4+ and CD8+ cells (Fig. 1B). The activation method (CD3/CD28 beads, PHA, or aAPCs) did not however affect the CD4:CD8 ratio for transduced cells (not shown). In addition, culture in either huIL-2 or caIL-2 did not affect the ratio (not shown).

However, we found that CAR T cells expanded with rhIL-2 had statistically superior transduction efficiency, with a median transduction efficiency of 16.3% compared to 5.07% median transduction efficiency for rcaIL-2 cultured CAR T cells (Fig. 1C). The method of activating the T cells also affected transduction efficiency, with activation with CD3/CD28 conjugated beads giving transduction efficiency of 37.2%, compared to 16.3% for aAPCs. T cells activated with CD3/CD28 conjugated beads had less variance between samples in terms of numbers of CAR T cells generated, with standard error of the mean (SEM) of 6.04 x 10^5^ compared to a SEM of 1.76 x 10^6^ for aAPC activated CAR T cells (Fig. 1D). Culture with rcaIL-2 supported robust initial T cell expansion with a mean 8-fold increase in total T cells, but by day 10 in culture the number of total CAR T cells generated was significantly lower in cultures with caIL-2 than in cultures with huIL-2 **(**not shown**)**.

The next studies examined the impact of adding cytokines (rhIL-7 and rhIL-15) on CAR T cell functionality. The rationale for addition of rhIL-7 was to promote CAR T cell survival and reduce T cell apoptosis [64], while rhIL-15 was added to promote T cell survival and proliferation of CD8+ memory T cells [65]. We found that addition of rhIL-7 and rhIL-15 to CAR T cell cultures from day 1 of culture significantly increased the killing capacity (cytolytic activity) of canine B7-H3 CAR T cells compared to cultures expanded in only IL-2 and IL-21, using the canine Abrams OS target cell (*p* value = 0.05) (Fig. 1E). In addition, CAR T cells used in this assay were previously frozen, thus demonstrating that the canine CAR T cells could be frozen and thawed and retain functional activity (Supplemental Fig. 1). These studies thus identified optimal cell culture activation and expansion conditions for generating canine B7-H3 CAR T cells.

### Expression of B7-H3 by canine OS cell lines and OS tissue biopsies

**Fig. 2:**
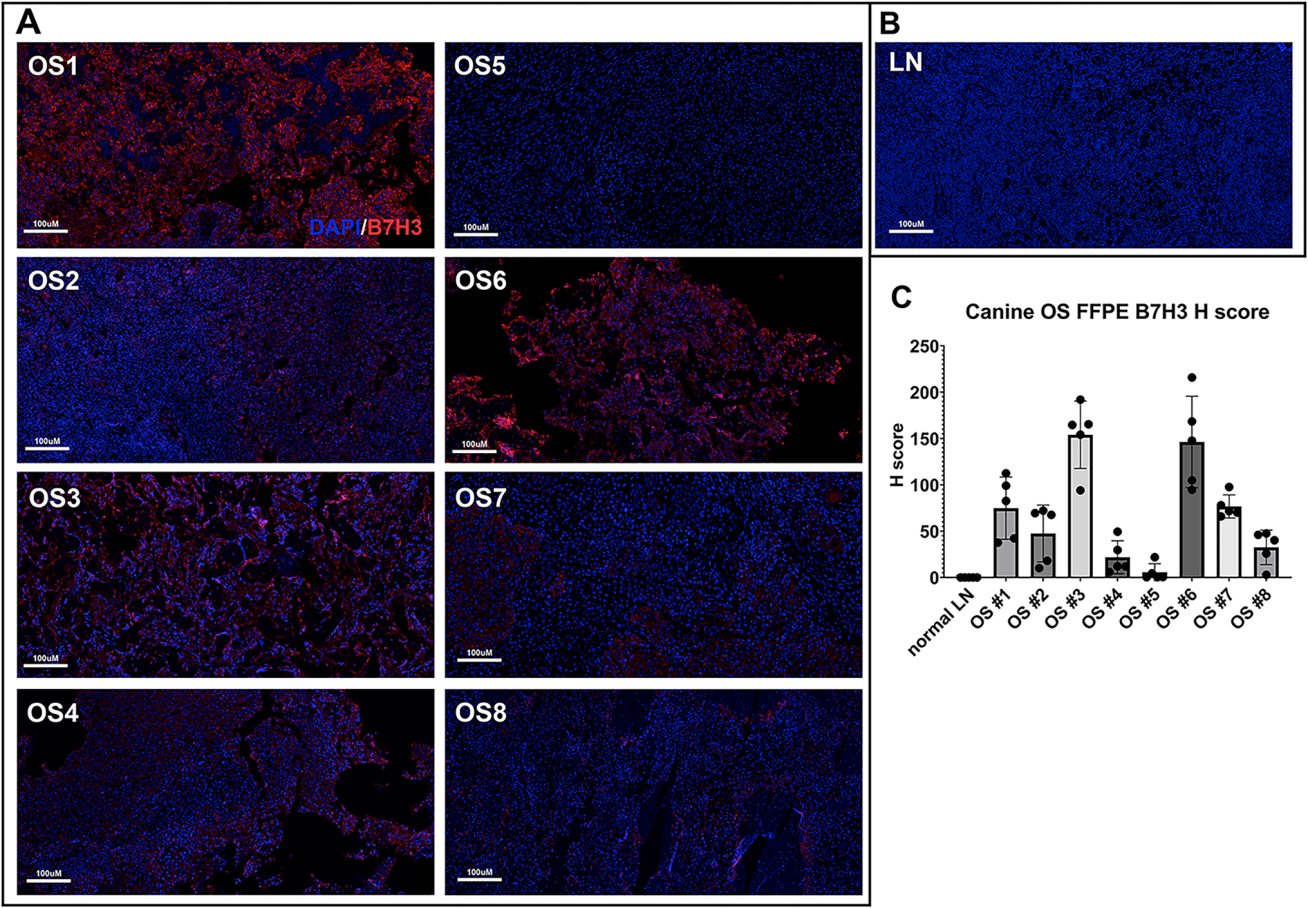
*Expression of B7-H3 by canine OS tumor tissues*. Tumor biopsies from n = 8 different dogs with primary, appendicular OS tumors were assessed to determine the relative levels of B7-H3 expression In **A**, tissues were immunostained for B7-H3 expression (red) using a cross-reactive mAb, and expression compared to immunostaining with an irrelevant mAb. In **B** an image of normal dog lymph node showed negative staining of B7H3. In **C**, the relative levels of B7-H3 expression by each of these 8 tumor biopsies were determined by ImageJ software (using 5 random 20 × magnification areas) to calculate an H-score, and expression was compared to that of normal canine lymph node (LN) tissue

Expanding on previous studies, [39] we evaluated the expression level of B7H3 in 7 canine OS cell lines and 8 tumor biopsies. These studies also included evaluation of B7-H3 expression by tissues from healthy animals (lymph node, spleen, and liver) (Supplementary Figure 2). This analysis revealed that all 8 OS tumor biopsy samples had positive B7-H3 expression, with expression levels above those exhibited by normal lymph node tissue (Fig. 2A, B). There was a range of expression levels, including three biopsies with strong B7-H3 expression, two with moderate expression and three with relatively low B7-H3 expression (Fig. 2C). In addition, analysis revealed minimal B7-H3 expression by healthy liver and spleen (Supplementary Figure 2). These findings therefore indicated that the high likelihood of B7-H3 expression by canine OS tumors made them suitable targets for treatment with canine B7-H3 CAR T cells, with low likelihood of on-target effects on normal tissues in dogs.

**Fig. 3:**
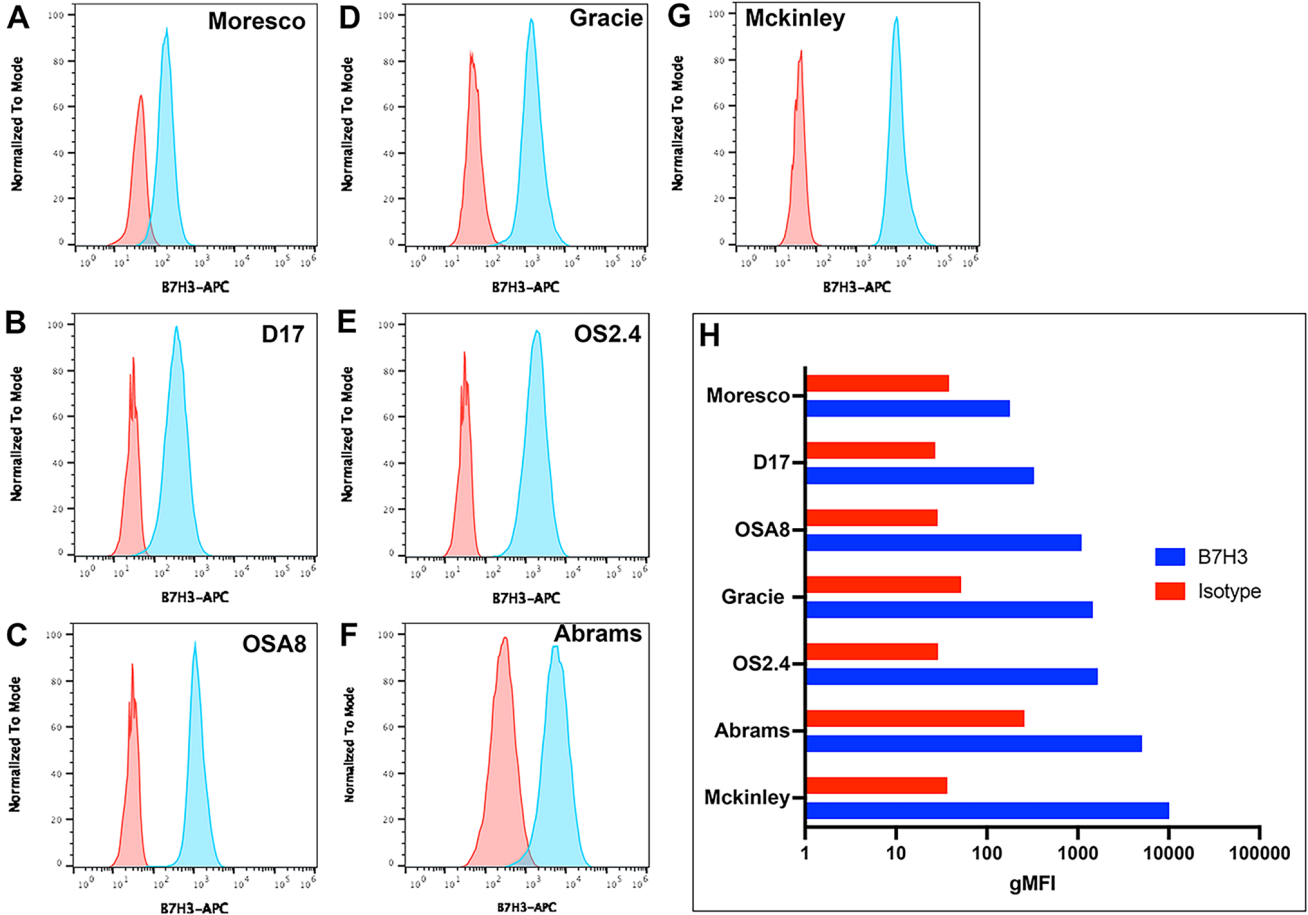
*Variable B7-H3 expression by 7 different canine OS cell lines*. The potential variability in OS cell line expression of B7-H3 was assessed using cell lines generated from 7 different canine OS tumors (as noted by either animal name, or a numeric designation). **A**–**G** depicts flow staining plots comparing B7H3 expression (blue histograms) to mouse IgG1 isotype control immunostaining (red histograms) for each cell line labeled in top right. H) Geometric mean MFI (mean florescence intensity) on log scale for each cell line B7H3 expression and corresponding isotype control staining in red

Seven canine OS cell lines were evaluated for surface expression of B7-H3 by flow cytometry (Fig. 3A–G). Cross reactivity of the mouse anti-human B7-H3 antibody (clone 7-517) with canine B7-H3 was first verified by western blot using canine Abrams OS cell line and human rhabdomyosarcoma cell line RH30 as positive controls (Supplementary figure 3). We found that all 7 cell lines were B7-H3 positive by flow cytometry, with 5 cell lines expressing high levels of B7-H3 expression and two OS lines exhibiting lower expression (Fig. 3H). Expression of B7-H3 by circulating canine leukocytes was also assessed, and it was determined that B7-H3 was expressed primarily by monocytes, though at very low levels (Supplementary Figure 4).

### Target engagement and functional activity of canine CAR T cells

**Fig. 4:**
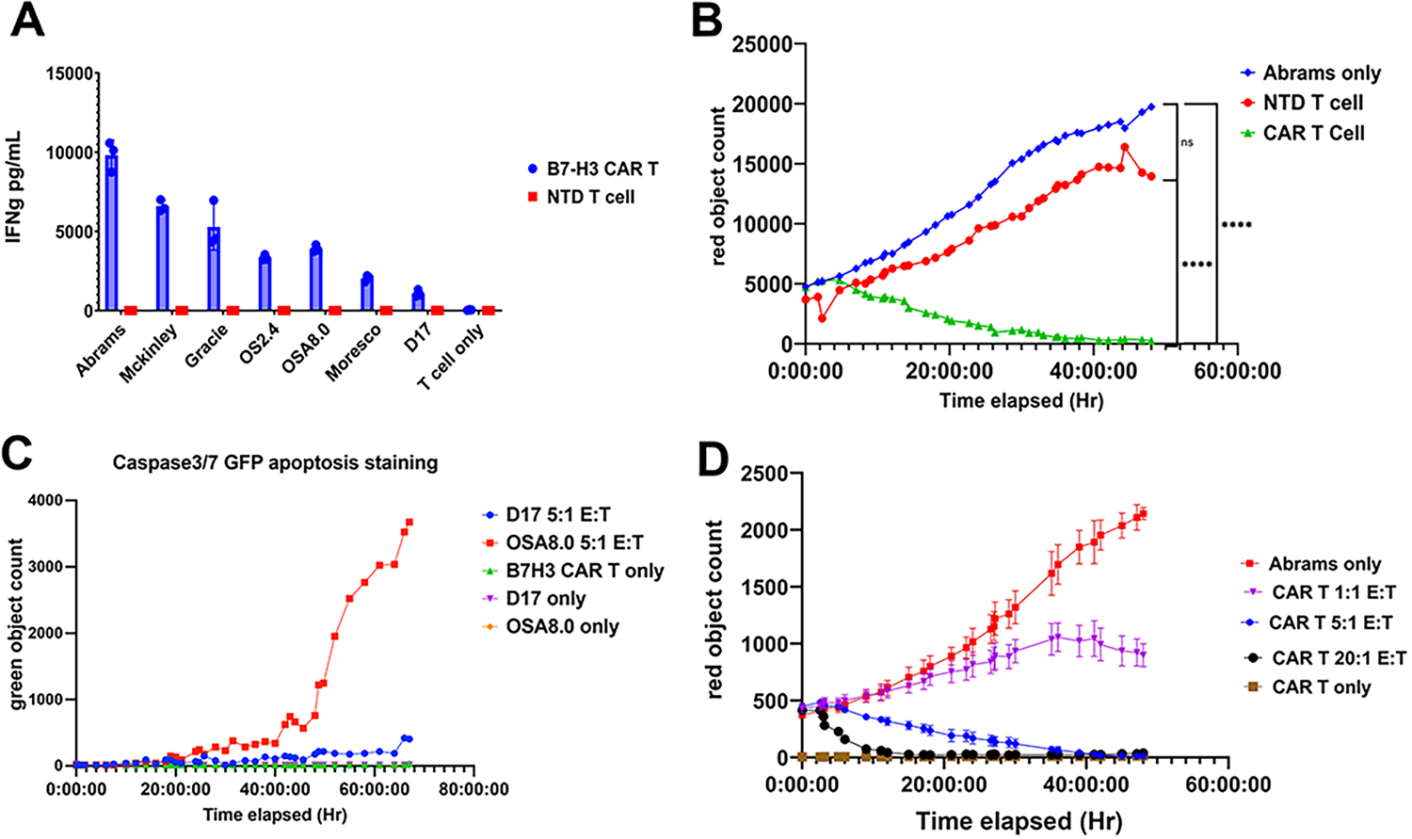
*Functional assessment of canine B7-H3 CAR T cells *in vitro*.* Canine B7-H3 CAR T cells were evaluated for their functional properties, including cytokine production and tumor cytotoxicity. In **A**, B7-H3 CAR T cells were cultured with 7 canine OS cell line targets, and production of IFN-γ was measured at 24 h. Cytokine production by non-transduced (NTD) T cells co-cultured with OS cell lines was included as a control. y-axis shows IFNg production in pg/mL. In **B**, killing of Abrams OS target cells by triplicate cultures of B7-H3 CAR T cells or NTD T cells was recorded over time using an Incucyte instrument, using an E:T ratio of 20:1 (effector CAR T to target cells) Y-axis shows total cell count (RFP red) with time elapsed on x-axis. Color legend shown in top right corner. In **C**, a different measure of cell killing (apoptosis induction by caspase 3 and 7 expression) was used to assess cytotoxicity of B7-H3 CAR T cells against either OSA8 target cells (B7-H3 high expressor), or D17 (B7-H3 low expressor) target cells. Y-axis shows total green (GFP- caspase) per image. In D), the impact of E:T ratios on target killing by B7-H3 CAR T cells was assessed, using Abrams OS cells as targets, at E:T ratios of 1:1, 5:1, and 20:1. Killing is depicted graphically as mean ± SEM**.** Statistical differences were determined by Dunn’s multiple means comparison, with **** = p < 0.0001 and ns = not significant

We next assessed the functional activity of canine B7-H3 CAR T cells, using canine OS cell lines as targets. To address this, canine B7-H3+ OS cell lines were co-cultured with canine B7-H3 CAR T cells at a 1:1 ratio and secreted IFN-γ was measured after 24 hours of incubation. We found that co-culture with B7-H3^high^ canine OS cell lines such as Abrams and McKinley triggered greater secretion of IFNγ by B7-H3 CAR T cells compared to co-culture with B7-H3^low^ cell lines such as Moresco and D17 (Fig. 4A). We also did not observe spontaneous IFNγ production by non-transduced T cells (NTD T Cells), indicating that T cell activation was occurring through interaction with the B7-H3 CAR (Fig. 4A).

As a second measure of target engagement and activation, we also measured tumor cytotoxicity of B7-H3 CAR T cells when cultured with the B7-H3^high^ Abrams OS cell line, using an Incucyte assay as described in Methods. We found that B7-H3 CAR T cells efficiently lysed Abrams OS cells, while NTD T cells did not exhibit significant lytic activity at the same E:T ratio (Fig. 4B). To further validate the target specificity of B7-H3 killing, we utilized an apoptotic marker caspase3/7-GFP to compare killing activity and found significant target killing of the B7-H3^high^ cell line (OSA8.0), but less killing of the B7-H3^low^ cell line (D17) (Fig. 4C). Additionally, B7-H3 CAR T cells demonstrated dose-dependent killing of Abrams-RFP canine OS cells with increased target cell lysis corresponding to an increased E: T ratio (Fig. 4D). These findings indicated therefore that canine B7-H3 CAR T cells recognized B7-H3 expressed by canine OS tumor cells, and exhibited both cytokine secretion and tumor cell lysis, in a CAR T cell dose-dependent and B7-H3 expression-dependent manner.

### Functional impact of co-expression of CXCR2 on CAR T cells

**Fig. 5:**
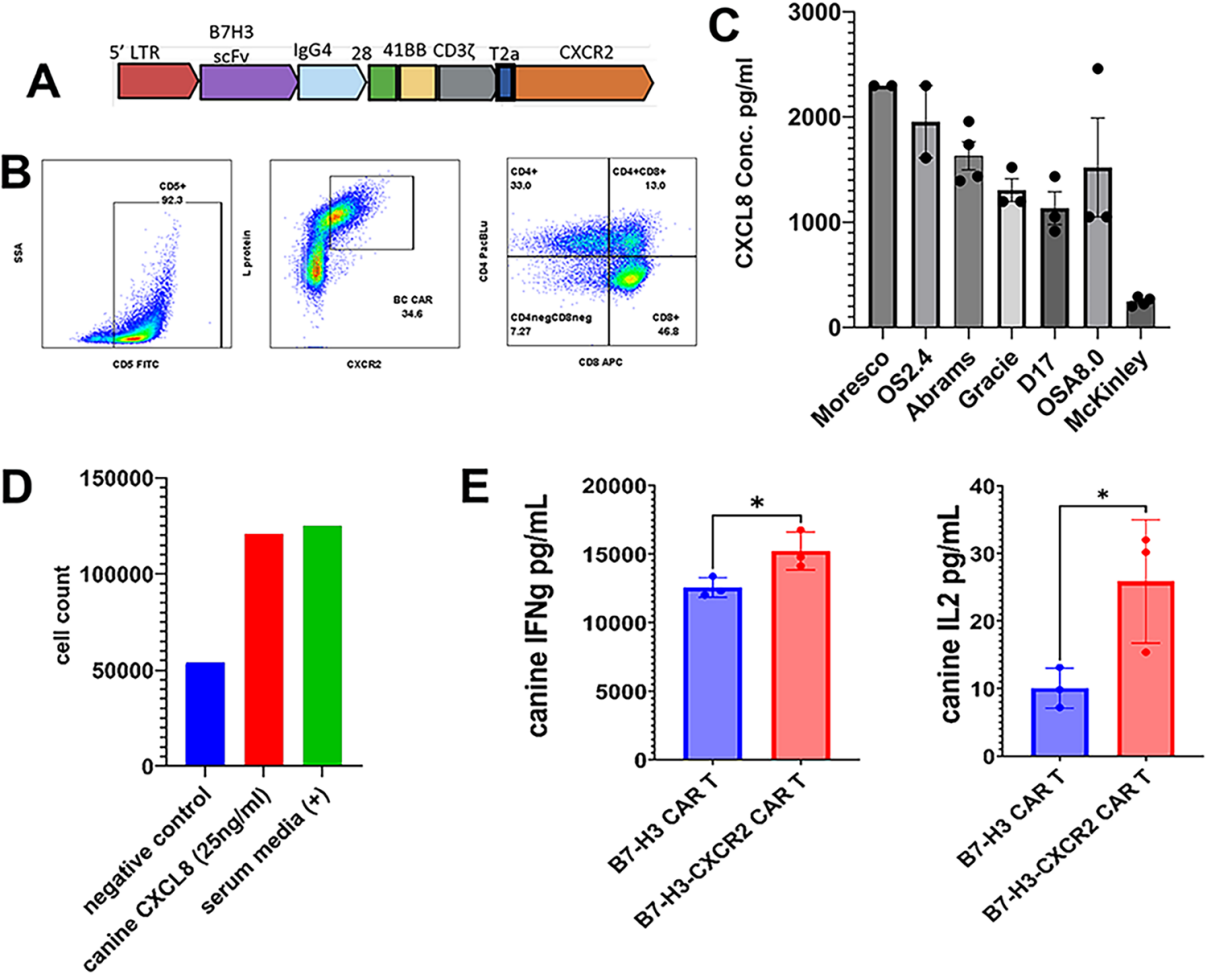
*Functional *in vitro* activity of dual B7-H3-CXCR2 CAR T cells compared to single CAR B7-H3 CAR T cells*. The ability of a chemokine receptor (CXCR2) to improve the activity of canine B7-H3 CAR T cells when the two molecules were co-expressed by transduced T cells was examined using in vitro studies. In **A**, the diagram of the dual B7-H3-CXCR2 construct is depicted. In **B**, the transduction efficiency of canine T cells with the dual CAR construct was evaluated by measuring L-protein binding (scFv detection) and with anti-huCXCR2 immunostaining, demonstrating expression of both molecules, along with the distribution of CD4 and CD8 CAR T cells. Flow cytometry dot plots showing percentage positive for each population. In **C**, to assess tumor target expression of a key chemokine (CXCL8) ligand for CXCR2, secretion of CXCL8 by 7 different canine OS tumor cell lines was measured, demonstrating that 6 of the 7 lines secreted large amounts of CXCL8 spontaneously. The ability of the huCXCR2 molecule to bind and become activated by canine CXCL8 was confirmed in **D**, using a transwell migration assay as described in Methods, with sustained migration of B7-H3-CXCR2 CAR T cells to a canine CXCL8 gradient. Serum containing medium was used as a positive control. In **E**, cytokine secretion following target Abrams cell engagement for 24 h was assessed, and revealed significantly greater production of both IFN-g and IL-2 with B7-H3-CXCR2 CAR T cells compared to B7-H3 CAR T cells, at equivalent E:T ratios (20:1)

Restricted infiltration into solid cancers such as sarcomas is considered a significant impediment to CAR T cell activity [5]. As one strategy to improve CAR T cell trafficking, the addition of a chemokine receptor such as CCR2 or CXCR2, has been reported to improve cell trafficking into tumors overexpressing chemokine ligands [42, 43]. Therefore, we evaluated the activity of a dual CAR construct, expressing both B7-H3 CAR and human CXCR2 (Fig. 5A), that had previously demonstrated superior activity in a rhabdomyosarcoma model [44]. To address this question, we generated canine B7-H3-CXCR2 CAR T cells and verified expression of both the B7-H3 CAR construct and CXCR2, as well as resulting CD4/CD8 T cell ratios (Fig. 5B).

We next assessed expression of the CXCR2 ligand CXCL8 by canine OS cells, using a canine CXCL8-specific ELISA (Fig. 5C). We found that 6 of 7 canine OS cell lines spontaneously produced large amounts of CXCL8, greater than 1000 pg/mL produced in 24 hours by 50,000 tumor cells on average (Fig. 5C). To determine the suitability of the human CXCR2 receptor for recruiting canine T cells, we tested whether B7-H3-CXCR2 CAR T cells could migrate to a gradient of canine CXCL8, using a transwell migration assay. These studies demonstrated that B7-H3-CXCR2 CAR T cells did in fact migrate toward a canine CXCL8 gradient (Fig. 5D).

Next, the impact of co-expression of CXCR2 on the function of CAR T cells was assessed. First, we observed that B7-H3-CXCR2 CAR T cells produced significantly more cytokines (IFNγ and IL-2) following tumor cell target engagement (Fig. 5E). While B7-H3-CXCR2 CAR T cells demonstrated increased cytokine production, their cytotoxic activity against canine OS targets was equivalent to that of B7-H3 CAR T cells (Supplemental Fig. 6)*.* It is also possible that target killing assays using adherent two-dimensional cultures may not fully reflect the true cytotoxic potential of CAR T cells, the Verneris laboratory (unpublished data) has shown that three-dimensional spheroid models are a closer approximation of *in vivo* tumors, and this model may more accurately demonstrate the CAR T penetration and killing potential. Thus, B7-H3-CXCR2 CAR T cells exhibited at least one measure of enhanced activity compared to single CAR T cells.

### B7-H3-CXCR2 and B7-H3 CAR T cells are transcriptionally distinct

**Fig. 6:**
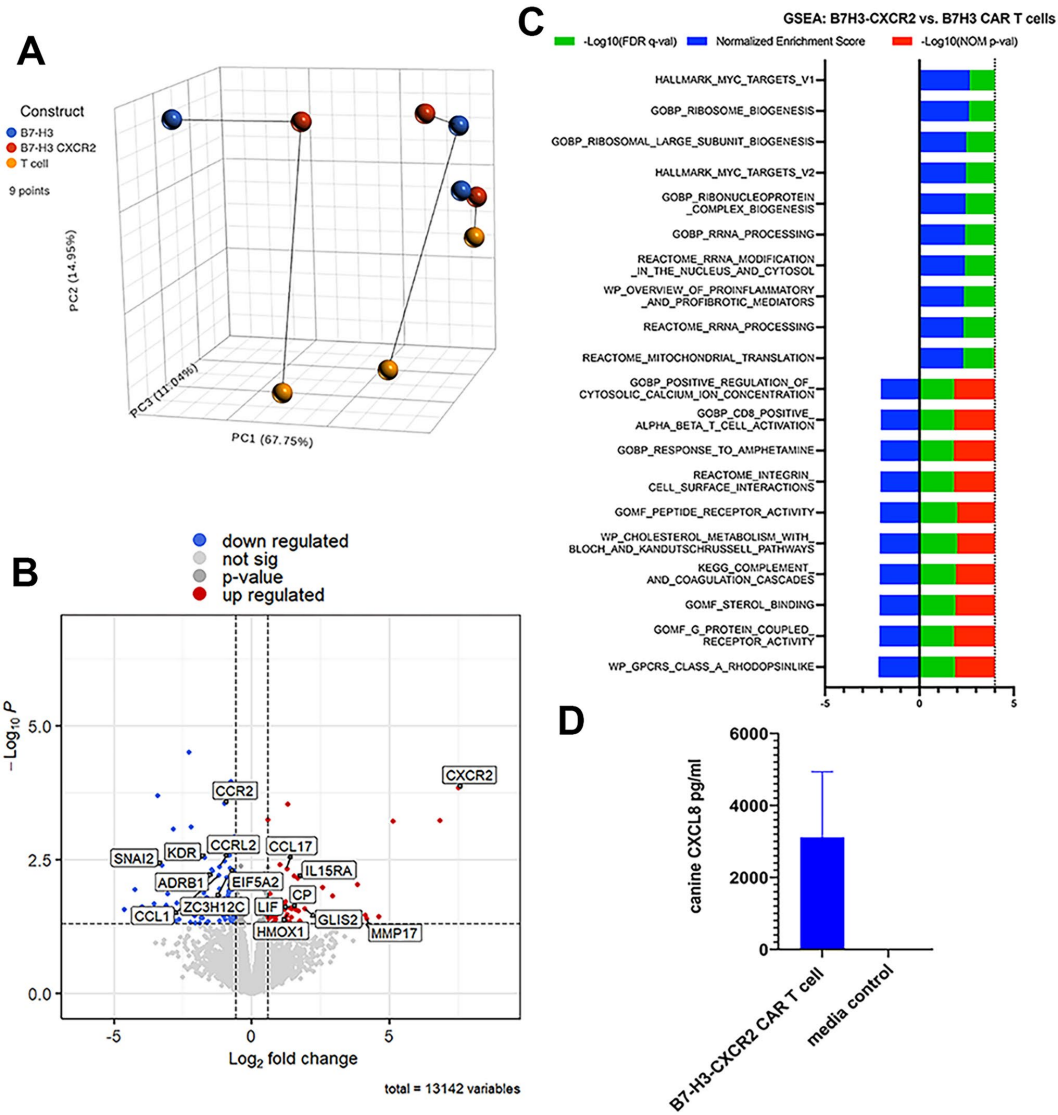
*Transcriptomic comparison of B7-H3 and B7-H3-CXCR2 CAR T cells*. RNA sequencing was performed to help elucidate in greater detail the functional differences between the two CAR T cells. In **A**, principal component analysis (PCA) plots depict B7-H3 CAR T cells, B7-H3-CXCR2 CAR T cells, and non-transduced T cells from 3 different dogs with biological replicates shown connected by lines. In **B**, volcano plots depict significantly upregulated (red) and downregulated (blue) genes in B7-H3-CXCR2 vs B7-H3 CAR T cells. Gene set enrichment analysis (GSEA) in **C** revealed significant upregulation (right of central axis) of pathways related to RNA processing and *myc* targets, along with downregulation of certain G-protein coupled receptor activities. Constitutive secretion of CXCL8 by B7-H3-CXCR2 CAR T cells in the absence of target cells was observed in panel **D**

To help elucidate mechanisms that might account for the increased activity of B7-H3-CXCR2 CAR T cells, we performed RNA sequencing on B7-H3 and B7-H3-CXCR2 CAR T cells, using cells generated from 3 individual dogs. These studies revealed transcriptional differences between the two different CAR T cells (Fig. 6A–C). For example, gene set enrichment analysis (GSEA) revealed multiple gene pathway differences (Figure 5C), including pathways associated with cell metabolism and respiration, receptor activity and activation (Table 1). To help explain why the B7-H3-CXCR2 CAR T cells might constitutively express upregulated activation of respiratory metabolic pathways, we examined the possibility that the CAR T cells were responding to CXCL8 produced by the CAR T cells themselves. Indeed, we found high concentrations of CXLC8 in the cultures of B7-H3-CXCR2 CAR T cells, in absence of tumor target cells (Fig. 6D). These findings suggested therefore the existence of an auto-stimulatory feedback loop, whereby CAR T cells responded to their own CXCL8 via CXCR2 signaling, leading to additional activation and production of more CXCL8.

### B7-H3-CXCR2 CAR T cells exhibit greater persistence and tumor control in a canine OS xenograft model

**Fig. 7:**
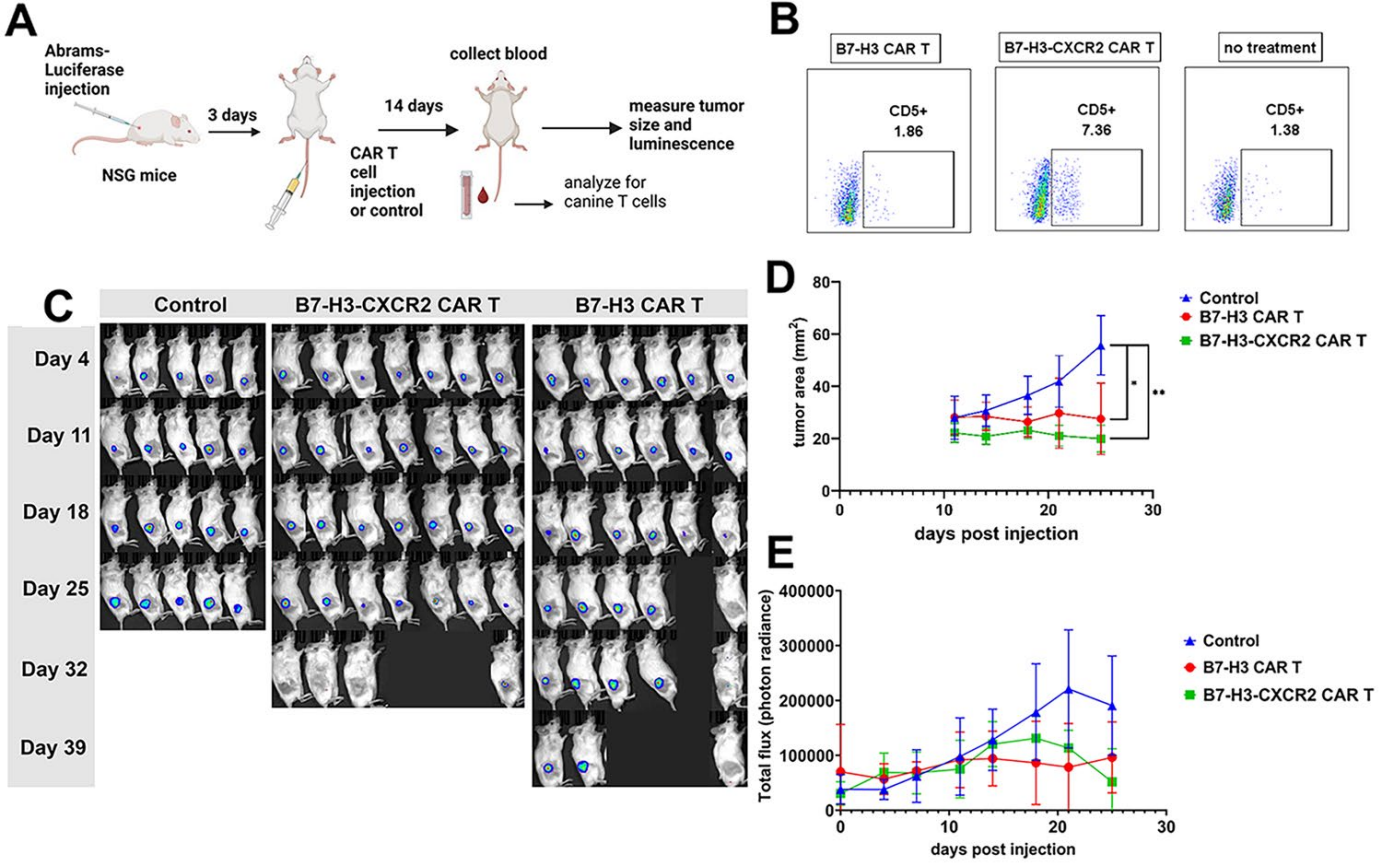
*Activity of B7-H3 and B7-H3-CXCR2 CAR T cells compared in canine OS xenograft model*. The relative ability of canine CAR T cells generated using the two different CAR constructs to control growth of a canine OS tumor (Abrams) in NSG mice was evaluated, with the study design illustrated in **A**. In **B**, the persistence of the two CAR T cells in blood of mice at day 14 was assessed using flow cytometric detection of canine CD5 T cells, revealing undetectable CAR T cells in B7-H3 CAR T injected mice; whereas, CAR T cells remained detectable in B7-H3-CXCR2 CAR T injected mice. Tumor responses to CAR T cell treatment were measured in **C**, using BLI, as noted in Methods, and revealed greater tumor control and elimination in mice treated with B7-H3-CXCR2 CAR T cells, compared to untreated mice or mice treated with B7-H3 CAR T cells. In **D**, tumor growth was quantitated using direct volume measurements (upper panel) or BLI measurements (lower panel) and revealed significantly greater tumor control in animals treated with B7-H3-CXCR2 CAR T cells. Data represent mean ± SEM of tumor area or photon flux, with * and ** reflecting statistical significance of *p* < 0.05 and p < 0.01 respectively, as determined by repeated measures ANOVA for tumor area

Finally, we conducted studies to determine whether expression of CXCR2 improved the anti-tumor activity of canine B7-H3 CAR T cells, using a mouse xenograft model of NSG mice implanted with canine OS cells. For these studies, we confirmed that the transduction efficiencies for the two CAR T cells were similar (Supplemental Fig. 7). First, we measured the numbers of B7-H3-CXCR2 and B7-H3 CAR T cells in the bloodstream of mice on day 14 after CAR administration (Fig. 7A,B). This analysis revealed that B7-H3-CXCR2 CAR T cells were still present in circulation two weeks after injection; whereas, B7-H3 CAR T cells were undetectable.

Next, the impact of CAR T cell treatment on tumor growth was measured, using bioluminescent imaging (BLI) imaging and direct caliper measurements. We found using BLI that mice treated with B7-H3-CXCR2 CAR T cells had significant inhibition of tumor growth, with 3 out of 7 mice experiencing complete tumor regression by day 39 after tumor injection. In contrast, mice treated with B7-H3 CAR T cells had one complete tumor regression, with slowed growth in the other 5 mice. Untreated control mice all had progressive growth of tumors and were euthanized on day 25 (Fig. 7C). It should also be noted that after day 39, all of the animals receiving either B7-H3-CXCR2 or B7-H3 CAR T cells began to die from graft-versus-host disease, as revealed by immunostaining of multiple tissues and by flow cytometric analysis, which revealed a dense infiltrate of canine CD5+ T cells, both CD4+ and CD8+ (data not shown). Physical measurement of tumor size indicated that mice treated with B7-H3 CAR T cells and B7-H3-CXCR2 CAR T cells had statistically smaller tumors compared to control mice, though tumor rates growth between CAR T cell treatment groups were not statistically different (Fig. 7D). Total photon flux as measured by BLI revealed that untreated animals had tumor photon flux increasing over time whereas in the mice treated with CAR T cells had stable photon flux through day 25 (Fig. 7E). These findings indicated therefore that B7-H3-CXCR2 CAR T cells exhibited greater persistence and anti-tumor activity than B7-H3 CAR T cells and indicated that CXCR2 signaling contributed multiple beneficial effects on overall CAR T cell function, not limited to only enhanced recruitment.

## Discussion

In this study, we provide evidence that canine B7-H3 CAR T cells can specifically kill B7-H3 expressing canine OS target cells, both *in vitro* and *in vivo*. In addition, we demonstrated that co-expressing a chemokine receptor (CXCR2) with the CAR construct significantly improved overall canine CAR T cell anti-tumor activity, consistent with our previous work demonstrating enhanced human CAR T cell activity against rhabdomyosarcoma [44] . Importantly from the standpoint of dogs as a model for evaluating B7-H3 CAR T cells, we found negligible B7-H3 expression by normal dog tissues, in contrast to generally high but variable expression by canine OS tumor tissues. First trials evaluating CD19 and CD20 CAR T cells in canine lymphoma [48, 66] showed promising results in that there was limited dose related toxicity and absence of CRS (cytokine release syndrome) which is a major concern in human clinical trials [67]. However, in these canine trials elimination of tumor target was transient and there were no circulating CAR T cells found at the low doses administered up to 72 hours.

We first optimized protocols for generating canine CAR T cells, which involved in part comparing three different methods of T cell activation, and two different cytokine protocols for CAR T cell expansion. We found that in agreement with a previously published protocol [51, 63], T cell activation using conjugated canine anti-CD3/CD28 beads provided the best overall CAR T cell expansion stimulus, with the least variability between biological samples (Fig. 1D). In addition, we found that culture with rhIL-7 and rhIL-15 improved CAR T cell cytotoxic activity (Fig. 1E) [48, 51]. Using this optimized protocol, we were also able to efficiently generate CAR T cells from blood of dogs with osteosarcoma, thus validating the approach for clinical studies in dogs with cancer. The transfection efficiency also matched previously reported B7H3 canine CAR T transduction efficiencies [39] though whereas the Zhang et al. study utilized blood from healthy dogs, our study used blood from dogs with sarcoma in order to more accurately reflect the clinical setting for autologous CAR T cell therapy.

In a previous clinical trial with B7-H3 CAR T cells for glioma, the intraventricular route was used to deliver the CAR T cells directly to the CNS [68]. Although the CAR T cells remained in the CSF for up to 4 weeks, only one out of 4 of the patients showed sustained clinical improvements. In a second clinical trial, a patient was implanted with a delivery device to allow direct CAR T cell injection into brain tumor tissues [69]. However, in this study it was reported that the CAR T cells did not reach the tumors regions that were furthest from the delivery point. Therefore, to help improve tumor trafficking to tumor tissues, in the studies reported here we also evaluated the impact of addition of a chemokine receptor (CXCR2) to the CAR construct, to enhance trafficking to a CXCL8 gradient, which is present in many tumor types. The use of this dual CAR construct has been reported previously by some of our group (JL, MV) to significantly improve the functionality of B7-H3 CAR T cells in human xenograft models [44].

Indeed, we found that expression of the huCXCR2 molecule in canine CAR T cells resulted in enhanced functionality *in vitro*, as revealed by significantly increased cytokine release (Fig. 5E; Supplemental Fig. 6) and upregulation of genes related to T cell activation (e.g., ZIRC2 and IL-15A). We also found that the B7-H3-CXCR2 CAR T cells were superior to B7-H3 CAR T cells in their ability to persist *in vivo* (Fig. 7B) and to eradicate tumors (Fig. 7C). These studies together therefore provided evidence that B7-H3-CXCR2 CAR T cells were more functionally active than the single B7-H3 CAR T cells and provided new insights into potential mechanisms underlying this increased activity.

There were several limitations to the studies reported here. In particular, the effector and memory phenotypes of CAR T cells were not assessed, and this information would have been helpful in assessing their ability to persist and to exert sustained effector functions. Investigating T cell epigenetic changes and the impact of addition of CXCR2 expression could have provided additional insights into mechanisms underlying improved CAR T cell function. Lastly, wider dose-ranging studies might have been able to reveal greater differences in the anti-tumor activity of B7-H3-CXCR2 and B7-H3 CAR T cells. For future clinical studies, it is also worth exploring the addition of TME modifying drugs to deplete immune suppressive myeloid cell populations [70], thus converting it into an environment more favorable to T cells, as we have reported recently for osteosarcoma and glioma in dogs [54, 71].

In summary, we developed optimized CAR T cell protocols for generating canine B7-H3 CAR T cells and demonstrated the improved anti-tumor activity of dual CAR T cells expressing the B7-H3 CAR and the chemokine receptor CXCR2, in agreement with previous studies evaluating this construct in human CAR T cells. We also found that autocrine signaling via CXCR2 and CXCL8 (and potentially other CXCR2 chemokine ligands) produced by the CAR T cells themselves might account in part for increased constitutive activity of the B7-H3-CXCR2 CAR T cells. Thus, our findings help provide additional validation for the concept of pairing chemokine receptors with targeting CAR constructs, to improve both intrinsic T cell functionality and anti-tumor activity. By evaluating these novel CAR constructs in canine spontaneous OS models, it should be possible to advance these CAR T cells more efficiently to pediatric trials.

### Supplementary Information

Below is the link to the electronic supplementary material.

Supplementary file 1 Supplemental Figure 1. Impact of cell freezing on CAR T cell recovery and CAR expression. B7-H3 CAR T cells were subjected to cell freezing after day 7 of culture, as described in Methods, and then cultured for an additional 7 days after thawing in either IL-2 alone or IL-2 + IL-7 and IL-15. The percentage of CAR TT cells in each of the two culture conditions was determined using L-protein binding and flow cytometry and revealed high CAR T cell recovery in both conditions. Supplemental Figure 2. Expression of B7-H3 by normal canine tissues. Liver and spleen tissues from a healthy dog were immunostained with an anti-B7-H3 mAb and imaged by confocal microscopy. These studies revealed very low levels of expression in the spleen, and undetectable expression by liver tissues. Supplemental Figure 3. Western blot to demonstrate specificity of B7-H3 mAb for recognition of canine B7-H3. The anti-human B7-H3 mAb used in these studies was used for Western blotting of lysates generated from a human rhabdomyosarcoma line (RH30) and a canine OS line (Abrams). These blots revealed positive binding to a protein of the predicted molecular size of human B7-H3 (55kDa), as well as a second smaller protein of unknown origin. Supplemental Figure 4. B7H3 expression by circulating leukocytes in dogs. Blood from a healthy dog was prepared by Ficoll density centrifugation, and peripheral blood mononuclear cells (PBMC) were immunostained with the anti-human B7-H3 mAb and with antibodies to CD5 (T cells), CD21 (B cells) and monocytes (CD11b, CD14), which revealed expression only by circulating monocytes (see dot plot, bottom left panel). Supplemental Figure 6. Relative tumor cytotoxicity of B7-H3 versus B7-H3-CXCR2 CAR T cells. The relative abilities of B7-H3 and B7-H3-CXCR2 CAR T cells to lyse canine Abrams OS cells at differing E:T ratios were assessed using an Incucyte assay. Supplemental Figure 7. Transduction efficiency of B7-H3-CXCR2 (BC CAR) and B7-H3 CAR T cells used in canine OS xenograft model. The transduction efficiencies of the two CAR constructs used in the xenograft model (Figure 7) were similar, each at approximately 22% CAR T cells. (PDF 1000 kb)
