# Supplementary material for: Targeting osteosarcoma with canine B7-H3 CAR T cells and impact of CXCR2 Co-expression on functional activity
Source: Cancer Immunol Immunother. 2024 Mar 30;73(5):77. doi: 10.1007/s00262-024-03642-4 (PMC10981605; doi:10.1007/s00262-024-03642-4)
Supplement: Supplementary file 1 — Supplemental Figure 1. Impact of cell freezing on CAR T cell recovery and CAR expression. B7-H3 CAR T cells were subjected to cell freezing after day 7 of culture, as described in Methods, and then cultured for an additional 7 days after thawing in either IL-2 alone or IL-2 + IL-7 and IL-15. The percentage of CAR TT cells in each of the two culture conditions was determined using L-protein binding and flow cytometry and revealed high CAR T cell recovery in both conditions. Supplemental Figure 2. Expression of B7-H3 by normal canine tissues. Liver and spleen tissues from a healthy dog were immunostained with an anti-B7-H3 mAb and imaged by confocal microscopy. These studies revealed very low levels of expression in the spleen, and undetectable expression by liver tissues. Supplemental Figure 3. Western blot to demonstrate specificity of B7-H3 mAb for recognition of canine B7-H3. The anti-human B7-H3 mAb used in these studies was used for Western blotting of lysates generated from a human rhabdomyosarcoma line (RH30) and a canine OS line (Abrams). These blots revealed positive binding to a protein of the predicted molecular size of human B7-H3 (55kDa), as well as a second smaller protein of unknown origin. Supplemental Figure 4. B7H3 expression by circulating leukocytes in dogs. Blood from a healthy dog was prepared by Ficoll density centrifugation, and peripheral blood mononuclear cells (PBMC) were immunostained with the anti-human B7-H3 mAb and with antibodies to CD5 (T cells), CD21 (B cells) and monocytes (CD11b, CD14), which revealed expression only by circulating monocytes (see dot plot, bottom left panel). Supplemental Figure 6. Relative tumor cytotoxicity of B7-H3 versus B7-H3-CXCR2 CAR T cells. The relative abilities of B7-H3 and B7-H3-CXCR2 CAR T cells to lyse canine Abrams OS cells at differing E:T ratios were assessed using an Incucyte assay. Supplemental Figure 7. Transduction efficiency of B7-H3-CXCR2 (BC CAR) and B7-H3 CAR T cells us [file 262_2024_3642_MOESM1_ESM.pdf]

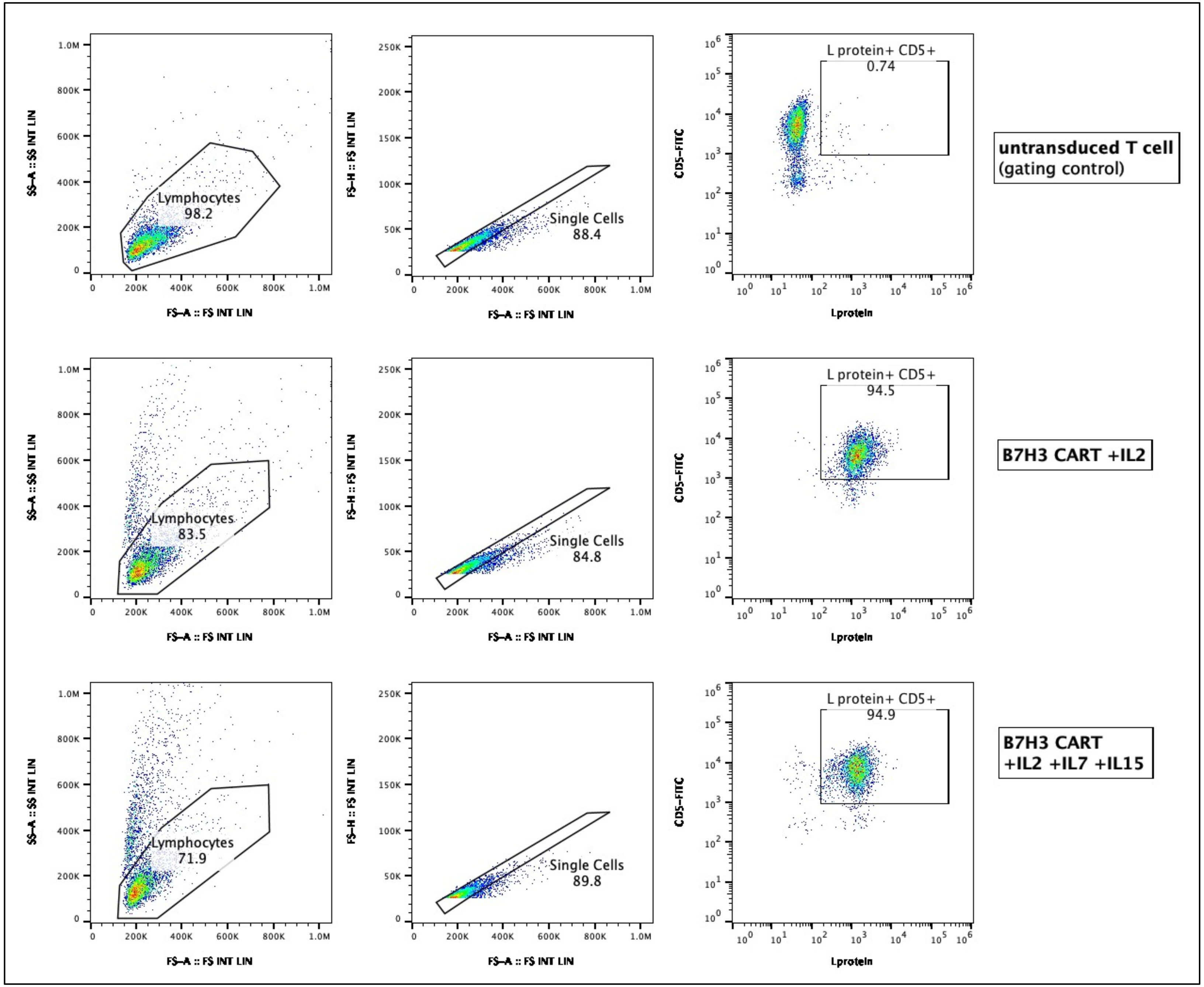

Supplemental Figure 1. expression of CAR of canine B7-H3 CAR T cells post freeze thaw cycle at day 14 cultured in either IL-2 or IL-2, IL-7 and IL-15

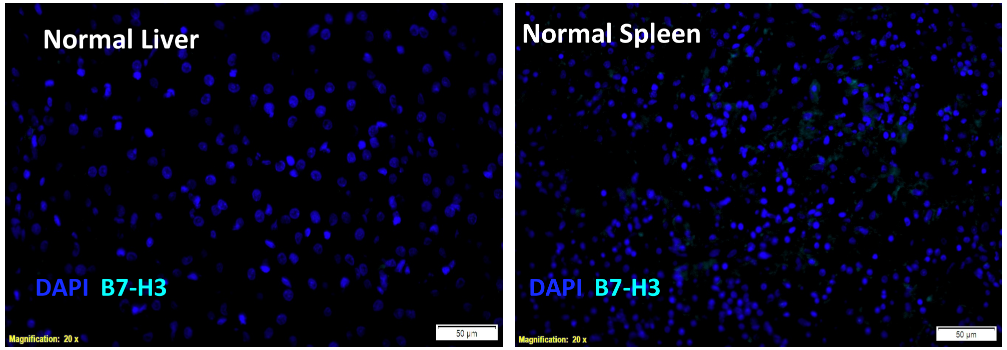

Supplemental Figure 2. expression of B7-H3 on Zinc Fixed normal canine tissues liver, spleen, and lymph node

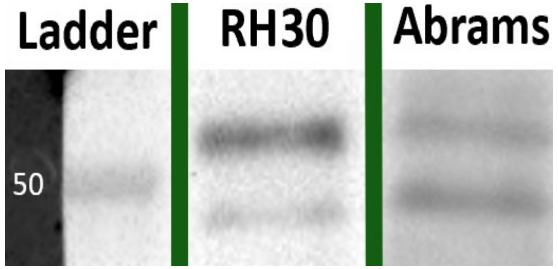

Supplemental Figure3. Human B7H3 monoclonal antibody binding to human rhabdosarcoma cell line (RH30) and canine OS cell line (Abrams) B7H3 protein by western blot at size 55kDA

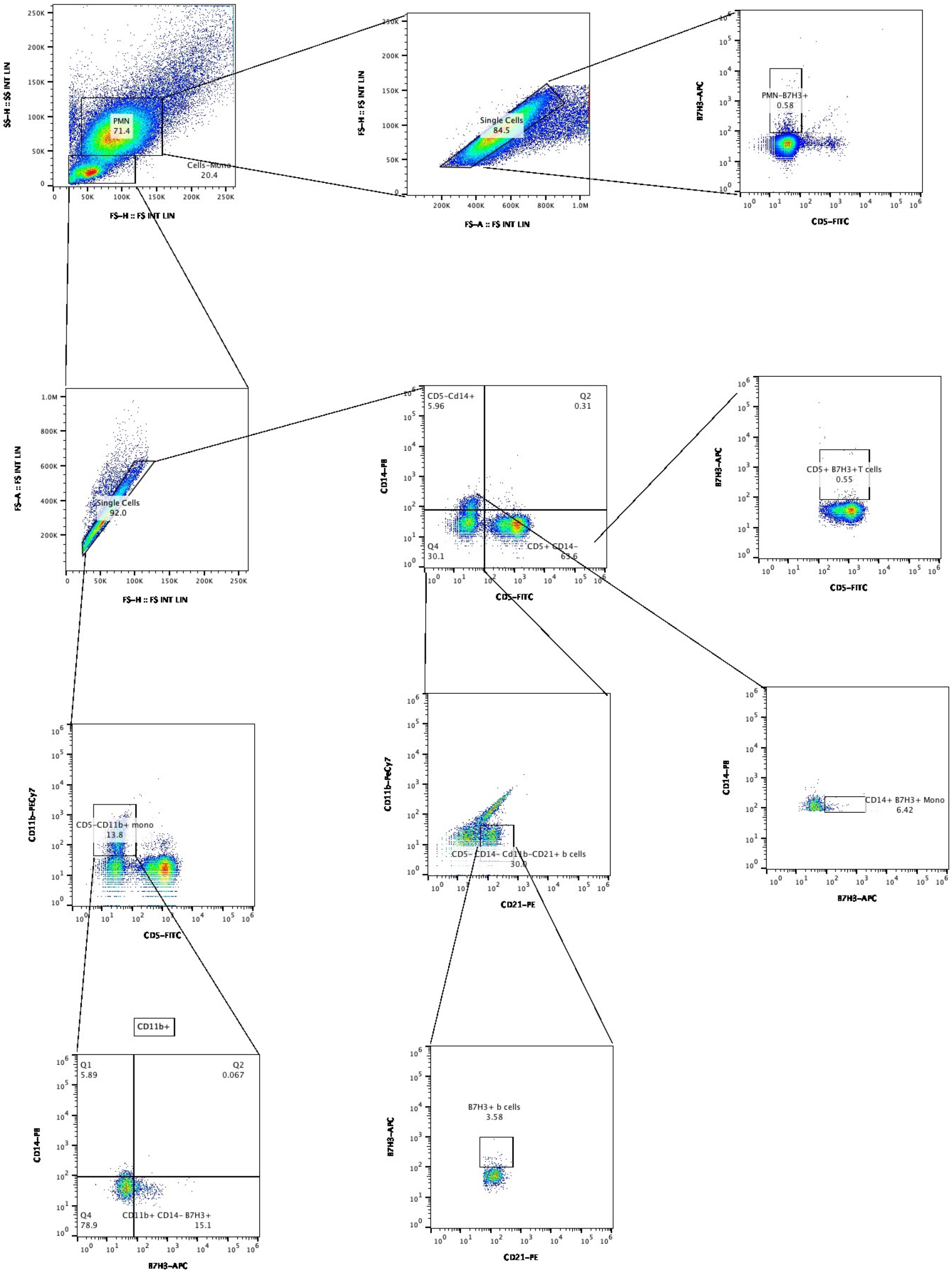

Supplemental Figure 4. B7H3 expression on healthy dog PBMC by flow cytometry

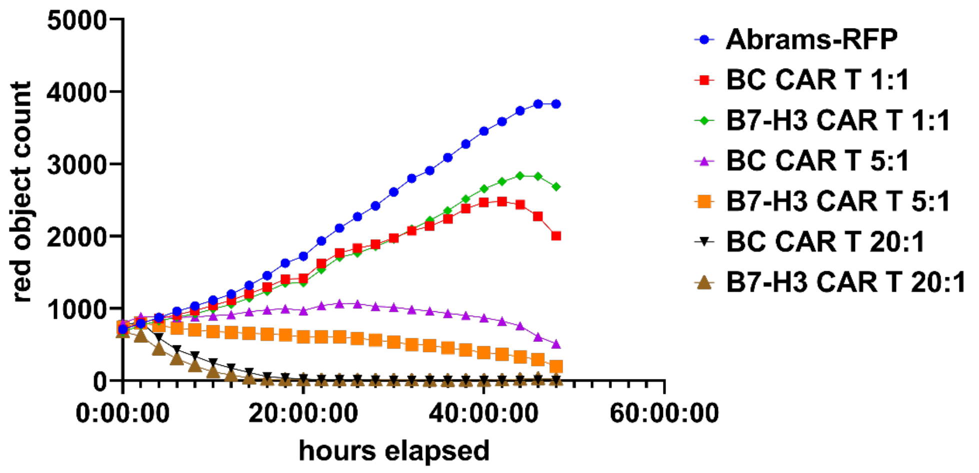

Supplemental Figure 5. *in vitro* killing of canine OS cell line Abrams RFP by donor matched B7-H3-CXCR2 CAR T cells (BC CART) or B7-H CAR T cells at effector: target (E:T) ratios 1:1, 5:1, and 20:1 over 48 hours

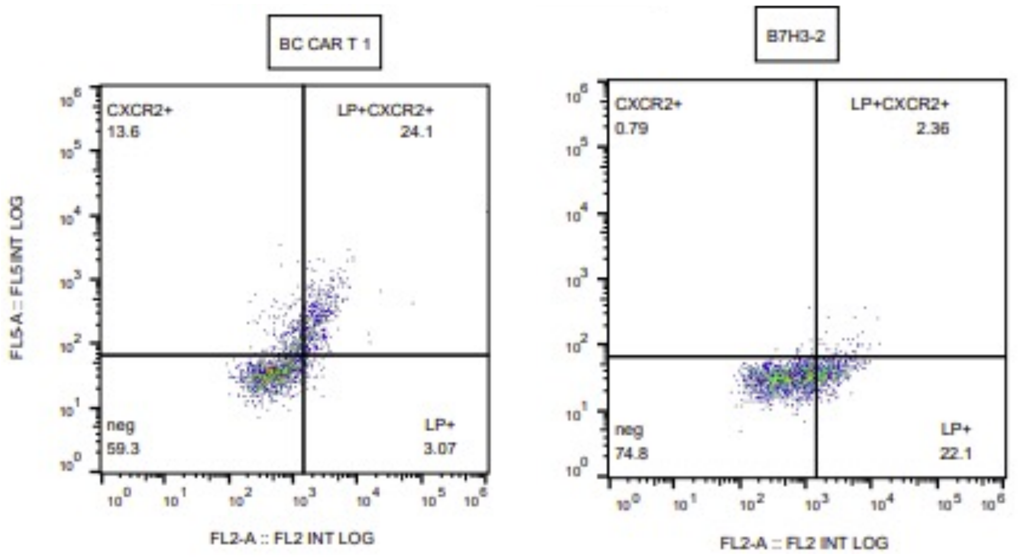

Supplemental Figure 6. Transduction efficiency of CAR T cells pre-injection of NSG mice
